# Prognostic value of various immune cells and Immunoscore in triple-negative breast cancer

**DOI:** 10.3389/fimmu.2023.1137561

**Published:** 2023-04-06

**Authors:** Xinyu Ren, Yu Song, Junyi Pang, Longyun Chen, Liangrui Zhou, Zhiyong Liang, Huanwen Wu

**Affiliations:** ^1^ Department of Pathology, State Key Laboratory of Complex Severe and Rare Disease, Molecular Pathology Research Center, Peking Union Medical College Hospital, Chinese Academy of Medical Sciences and Peking Union Medical College, Beijing, China; ^2^ Department of Breast Surgery, Peking Union Medical College Hospital, Chinese Academy of Medical Science and Peking Union Medical College, Beijing, China

**Keywords:** triple-negative breast cancer, immunohistochemical staining, immune microenvironment, image registration, Immunoscore

## Abstract

**Background:**

This study aimed to evaluate the expression status and prognostic role of various immunoregulatory cells and test in triple-negative breast cancer (TNBC).

**Methods:**

The expression of five markers (CD3/CD4/CD8/CD19/CD163) of tumor immune cells was evaluated retrospectively in tumor sections from 68 consecutive cases of TNBC by immunohistochemistry. Computational image analysis was used to quantify the density and distribution of each immune marker within the tumor region, tumor invasive margin, and expression hotspots. Immunoscores were calculated using an automated approach. Other clinical characteristics were also analyzed.

**Results:**

For all patients, Kaplan–Meier survival analysis showed that high CD3+ signals in the tumor region (disease-free survival (DFS), *P*=0.0014; overall survival (OS), *P=*0.0031) and total region (DFS, *P=*0.0014; OS, *P=*0.0031) were significantly associated with better survival. High CD4+ levels in the tumor region and total regions were significantly associated with better survival (*P<*0.05). For Hotspot analysis, CD3+ was associated with significantly better survival for all Top1, Top2, and Top3 densities (DFS and OS, *P<*0.05). High CD4+ levels were significantly associated with better prognosis for Top1 and Top3 densities (DFS and OS, *P<*0.05). For stage IIB and IIIC patients, CD3+ in the tumor region and all Top hotspots was found to be significantly correlated with survival (DFS and OS, *P<*0.05). CD4+ cells were significantly associated with survival in the tumor region, total region, and Top3 density (DFS, *P=*0.0213; OS, *P=*0.0728). CD8+ cells were significantly associated with survival in the invasive margin, Top2 density, and Top3 density. Spatial parameter analysis showed that high colocalization of tumor cells and immune cells (CD3+, CD4+, or CD8+) was significantly associated with patient survival.

**Conclusion:**

Computational image analysis is a reliable tool for evaluating the density and distribution of immune regulatory cells and for calculating the Immunoscore in TNBC. The Immunoscore retains its prognostic significance in TNBC later than IIB stage breast cancer. Future studies are required to confirm its potential to predict tumor responses to chemotherapy and immune therapy.

## 1 Introduction

Triple-negative breast cancer (TNBC) is highly heterogeneous with an abundance of infiltrated immune cells (1). It is now known that the tumor microenvironment plays a very important role in the prognosis of and therapeutic efficacy against breast cancer, especially TNBC (2, 3). Tumor-infiltrating lymphocytes (TILs) and macrophages distribution has been shown to be heterogeneous, even within the same tumor tissue (4–7). Moreover, accumulating evidence indicates that TILs evaluated in different intra-tumoral regions play distinct roles in disease prognosis and predicting treatment responses (8–10). Collectively, these data suggest that analysis of TILs and macrophages from different tumor subareas is necessary. Different types of immune cells from different locations in the tumor microenvironment have different effects on tumors’ behavior, and it is necessary to study the role of lymphocytes further to obtain a complete and comprehensive understanding of the immune microenvironment in cancer progression (11). We chose to analyze five immune markers (CD3+, CD4, CD8+, CD19+, and CD163) because they represent the main immune cell types (cytotoxic and regulatory T cells, B cells, and macrophages) and play key roles in tumor immunity. Some scientists have proposed immune scores to evaluate the tumor immune microenvironment (TIME) and predict treatment efficacy and disease prognosis (12). However, the usefulness of immune scores across different types of tumors requires more corroborating data.

The quantification of TILs has the potential to become a new tumor biomarker that can be used to easily evaluate the immunogenicity of a tumor. Therefore, pathologists have begun to devise standardized visual approaches to quantify TILs to predict therapy efficacy. Evaluation of the tumor microenvironment mainly depends on visual observation of hematoxylin and eosin (HE)-stained slides (13). The successful application of this assessment method requires extensive training of pathologists. However, despite successful standardization efforts, evaluation by the human eye is subjective and interpretations can vary greatly among individuals. Visual TIL estimation has limited precision and lacks the ability to evaluate more complex properties such as TIL distribution patterns. In such situations, computational imaging analysis techniques can be applied to evaluate and calculate immune cell numbers in tumors more objectively and accurately and can help pathologists reduce their workload and improve the precision and efficiency of immunohistochemistry (IHC) image evaluation.

In this study, we used an automated method of image registration and analysis to evaluate the expression and distribution of various immune indicators identified by IHC, and then analyzed their prognostic capacity for TNBC.

## 2 Materials and methods

### 2.1 Patients

Specimens from 68 TNBC cases, selected randomly from a non-consecutive series of breast cancer cases from the Department of Pathology, Peking Union Medical College Hospital, were used in our study. Patient information is listed in **Table 1**. Data were collected in two batches due to the arrangement of the IHC staining schedule, with 19 patients in the first batch and 49 patients in the second batch. Clinicopathological parameters, including age, tumor size, histological grade, histological subtype, and lymph node status, were also reviewed. The follow-up period for this retrospective study was from the date of curative surgery until March 31, 2019, with a median follow-up time of 95 months.

**Table 1:** Information of patients.

Total number of patients		68
Age	< 40	11
	– 50	18
	> 50	39
Stage	I	15
	II	31
	III	22
vGrade	2	18
	3	50
Distant metastasis		20
Recurrence		27
Death		18

### 2.2 Tissue construction

Formalin-fixed paraffin-embedded (FFPE) samples were collected for each patient, with FFPE tissue sections prepared at a thickness of 4 μm. Seven slides of serial sections were collected per sample and stained for CD3, CD4, CD8, CD19, CD163, and cytokeratin (AE1/AE3), as well as H&E staining in order. IHC was performed according to the manufacturer’s protocol using a Leica stainer (Leica Biosystems, Germany). ER, PR, Her-2, P53, Ki67 were stained at time of diagnosis as previously described (14). HER-2 (2+) cases were further subjected to fluorescent *in situ* hybridization (FISH) to confirm its negative. Details of the primary antibodies of CD3, CD4, CD8, CD19, CD163, AE1/AE3 and their respective optimizations are listed in **Supplementary Table 1**. After staining, all the slides were scanned with a KF-pro-400 (Ningbo, China) scanner under the same acquisition conditions, with a magnification of 40× (0.2 μm/pixel). The images were then transformed into digital image files.

### 2.3 Image registration and annotation

**Figure 1:**
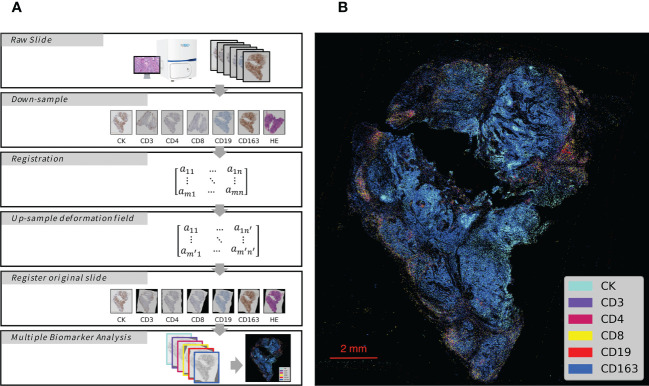
Schematic Overview of IHC Series Imaging, Registration and Multiplex Visualization. **(A)** Whole-Slide Digital Images representing serial sections enable assessment of immune microenvironment biomarkers. The images were down-sampled to a 500 pixels × 500 pixels size and then registered to the target images to generate deformation fields. The original size digital images were finally registered with up-sampled deformation fields, and thus serial sections identifying different biomarkers were spatially aligned to study the distributions of multiple immune cells. **(B)** Integrated visualization of serial sections by pseudo-coloring. Biomarkers and colors are shown on the right. The gray arrows indicated analytical steps that moves forward.

**Figure 2:**
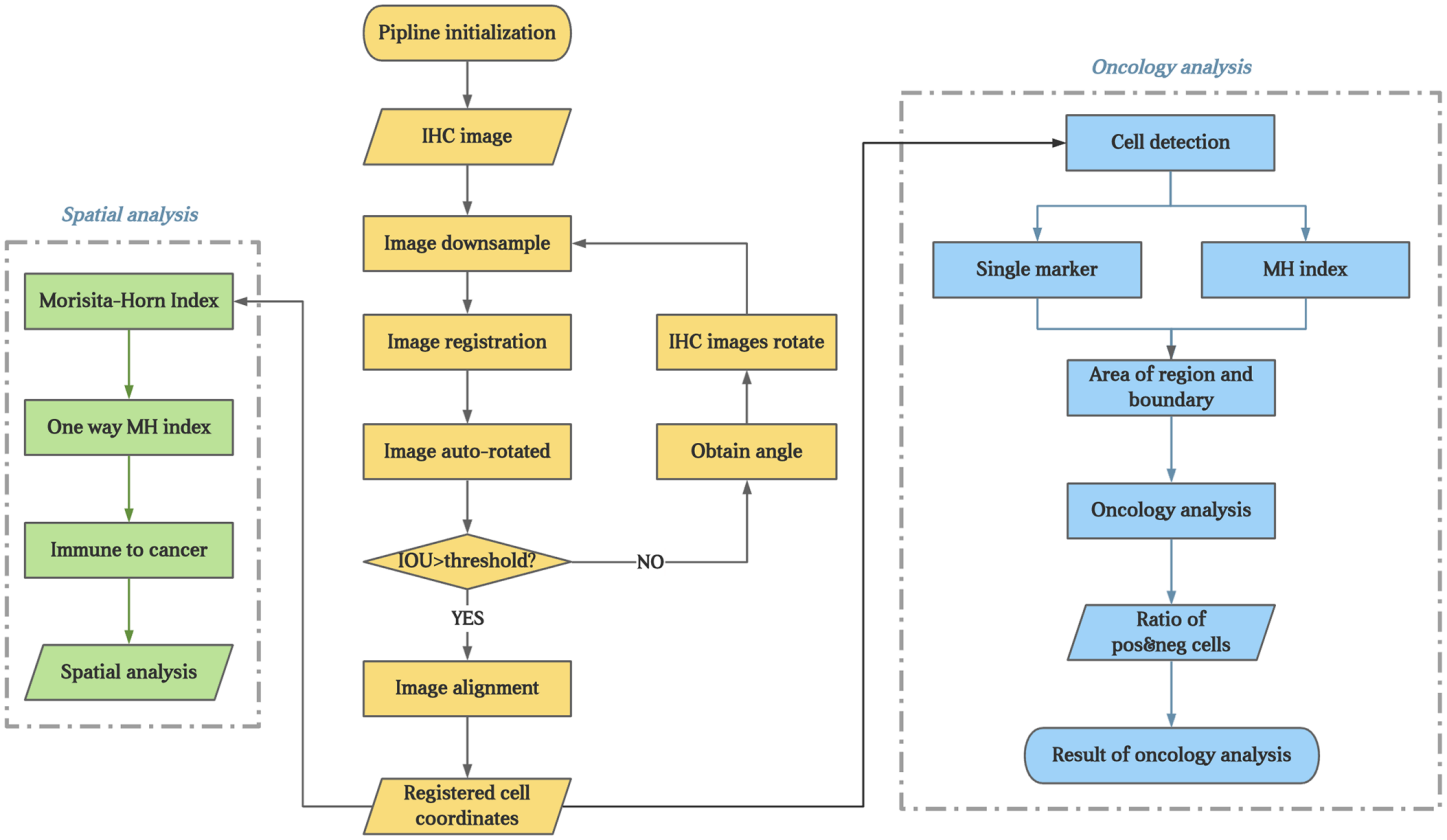
The flowchart of the experiment. The brown part present image registration and annotation process; The blue part present the analysis process of every IHC markers; The green part present process of colocalization of immune marker of tumor cells.

The process of analyzing the IHC images started with image registration to assess the spatial information among the biomarkers. The microscopic images were imported as digital slide files for image registration. To reduce errors, the digital slides were aligned to the fourth image, which was obtained from the middle slide of the serial sections. The typical registration process in this study is shown in **Figure 1A** (the upper 5 box), which was constructed and completed using Python. The images were first downsampled to a size of approximately 500 pixels × 500 pixels and then registered to downsampled target images with a step of rigid registration and a step of non-rigid registration. Deformation fields were generated and upsampled to the original image size. Finally, the digital image was registered with the upsampled deformation field, and serial sections identifying different biomarkers were spatially aligned to study the distributions of multiple immune cells (**Figure 1B**) with the whole process shown in **Figure 2**.

Owing to the different orders of serial sections in the two batches, the middle slides were CD8 for the first batch and CK for the second batch.

The tumor core (TC) and invasive front (IF) were annotated using QuPath by an experienced pathologist from the Department of Pathology, Peking Union Medical College Hospital. The invasive margin (IM) was defined as the region within 100 µm of the IF.

### 2.4 Quantification

For the IHC images, cell segmentation and quantification were assessed using QuPath (bule part of **Figure 2**). Cells positive and negative for each biomarker were measured with respect to quantity and location. Several parameters were measured for different biomarkers, as listed below.

#### 2.4.1 Quantification of single biomarker

For each biomarker, we calculated the positive cell density, which was defined as the positive cell number per mm^2^, in the TC and IM regions.

#### 2.4.2 Hotspot

Each slide image was dissected into small regions of 1000 pixels × 1000 pixels. A specific region was noted as an effective region if half of its area was in the annotated TC. Cell densities were calculated for every effective region and sorted from high to low. The top three positive cell densities were recorded as Hotspot1, Hotspot2, and Hotspot3 and used for further analysis.

#### 2.4.3 Colocalization

Colocalization was measured using the Morisita–Horn index (MH index), which is a parameter that indicates similarity among community structures in ecology. Mathematically, the MH index is defined as:


$$MH=\frac{2\sum_{i}p_{i}^{l}p_{i}^{c}}{\sum_{i}{\left(p_{i}^{l}\right)}^{2}+\sum_{i}{\left(p_{i}^{c}\right)}^{2}}$$

where $p_{i}^{l}$ and $p_{i}^{c}$ are the proportions of biomarkers *l* and *c*, respectively, within region *i*, and 1<*i*<R, where R is the total number of regions into which a sample image has been dissected. This equation can be expanded into multiple inputs. According to this equation, the MH index is between 0 and 1. The closer the MH index is to 1, the higher the colocalization of two or more biomarkers.

In addition, all immune cells were combined to calculate their density and MH index with tumor cells to determine the colocalization of immune marker of tumor cells (green part of **Figure 2**).

#### 2.4.4 Immunoscore

The Immunoscore was calculated using CD3+ and CD8+ cell densities in the TC and IM, as previously described (15). The CD3+ and CD8+ cell densities were converted into percentiles before calculating the Immunoscore as the average of four percentiles (two markers, two regions). A two-category Immunoscore analysis was applied, in which a density of 0–25% was scored as low, and a density >25% was scored as high.

### 2.5 Statistical analysis

All statistical analyses were performed in Python. For group comparison, all patients were separated into ER (early recurrence, patients with recurrence during the follow-up period) and LR (late recurrence, patients without recurrence during the follow-up period) groups, and DM (distant metastasis, patients with metastasis during the follow-up period) and non-DM (non-distant metastasis, patients without metastasis during the follow-up period) groups. A Student’s t-test was performed for each parameter, and a p-value<0.05 was regarded as significant.

For the prognostic analysis, disease-free survival (DFS) and overall survival (OS) were used as prognostic parameters. DFS and OS were calculated from the date of curative surgery to the date of first relapse or the date of death, respectively. Receiver operating curve (ROC) analysis was used to determine the valid cut-off point and the most discriminating threshold or cut-off value for each measurement. Univariate survival analysis was performed using the Kaplan–Meier method, and statistical differences were determined using the log-rank test. Multivariate survival analysis was performed using the Cox regression model. To study the influence of tumor stage on the prognostic analysis, patients with stages IIB and III cancer were separated from the group, and all analyses were repeated. A two-tailed p-value<0.05 was regarded as significant for all these analyses.

## 3 Results

### 3.1 Comparison of single biomarkers between groups

As shown in **Table 2**, the groups were compared for each measured parameter. The results showed that more than half of the parameters were significantly different between the ER and LR groups and the DM and non-DM groups. As can be seen from the results, for the 47 parameters measured in our study, twenty-five were significantly different between the ER and LR groups, and eighteen were significantly different between the DM and non-DM groups. There were eight parameters (cell density of CD19+ in TC, CD19+ in TC and IM, Hotspot2 of CD19+, Hotspot3 of CD19+, colocalization of CD3+ and CD8+, colocalization of CD4+ and CD163+, colocalization of CK+ and CD4+, and colocalization of CK+ and immune cells) with p-values<0.05 when comparing the ER and LR groups, but the corresponding p-values were >0.05 when comparing the DM and non-DM groups. However, only one parameter (Hotspot1 of CD8+) was significantly different when comparing the DM and non-DM groups, but not when comparing the ER and LR groups.

**Table 2:** The comparison of each calculated parameters (positive cell density) between the ER-LR groups and DM-non-DM groups.

Parameters	ER-LR p-value	DM-nonDM p-value	Parameters	ER-LR p-value	DM-nonDM p-value	Parameters	ER-LR p-value	DM-nonDM p-value
TC-CD3	0.002	0.002	Hotspot1-CD3	0.002	0.001	CD3-CD4	0.008	0.019
TC-CD4	0.004	0.014	Hotspot1-CD4	0.016	0.011	CD3-CD8	0.045	0.209
TC-CD8	0.076	0.104	Hotspot1-CD8	0.072	0.047	CD3-CD19	0.452	0.133
TC-CD19	0.021	0.166	Hotspot1-CD19	0.091	0.244	CD3-CD163	0.004	0.003
TC-CD163	0.078	0.052	Hotspot1-CD163	0.116	0.087	CD4-CD8	0.278	0.266
IM-CD3	0.004	0.001	Hotspot2-CD3	0.003	0.001	CD4-CD19	0.452	0.236
IM-CD4	0.004	0.003	Hotspot2-CD4	0.005	0.003	CD4-CD163	0.024	0.088
IM-CD8	0.101	0.056	Hotspot2-CD8	0.101	0.080	CD8-CD19	0.175	0.462
IM-CD19	0.072	0.195	Hotspot2-CD19	0.033	0.101	CD8-CD163	0.060	0.050
IM-CD163	0.050	0.026	Hotspot2-CD163	0.106	0.065	CD19-CD163	0.093	0.300
TC+IM-CD3	0.002	0.002	Hotspot3-CD3	0.002	0.001	CK-CD3	0.001	0.001
TC+IM-CD4	0.004	0.013	Hotspot3-CD4	0.003	0.004	CK-CD4	0.019	0.124
TC+IM-CD8	0.054	0.070	Hotspot3-CD8	0.132	0.111	CK-CD19	0.131	0.208
TC+IM-CD19	0.014	0.126	Hotspot3-CD19	0.026	0.071	CK-CD163	0.255	0.374
TC+IM-CD163	0.084	0.056	Hotspot3-CD163	0.103	0.068	CK-CD8	0.144	0.384
						Immunoscore	0.009	0.020

ER: early recurrence; LR: late recurrence; DM: distant metastasis; nonDM: non distant metastasis;

TC-CDx: The positive cell density of each biomarker in tumor core;

IM-CDx: The positive cell density of each biomarker in invasive margin;

TC+IM-CDx: The positive cell density of each biomarker in the region of tumor core and invasive margin together;

Hotspotx-CDx: The positive cell density of first, second and third hotspot for each biomarker;

CDx-CDx: The MH index between each pair of biomarkers; CK-CDx: The MH index between tumor cells and each biomarker.

### 3.2 Correlation between single biomarkers and prognostic survival

**Figure 3:**
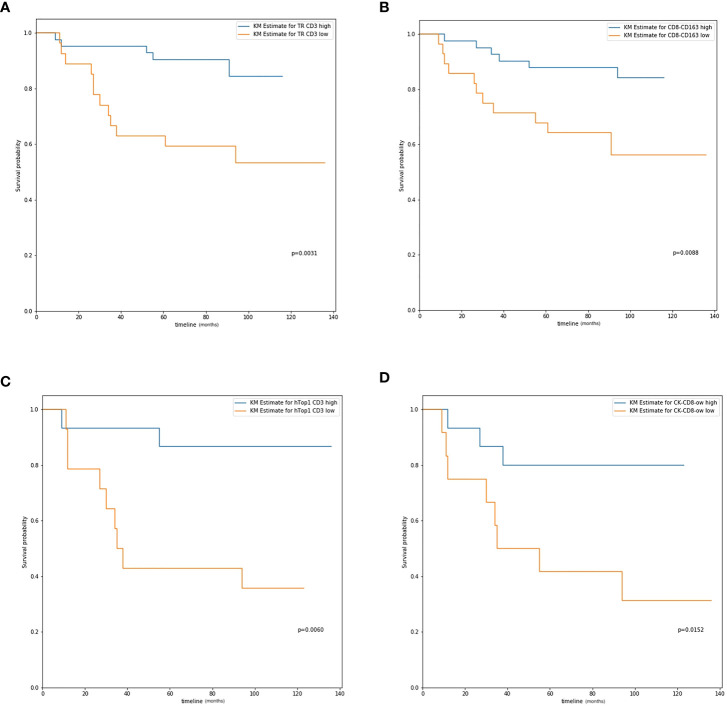
The overall survival curve. **(A)** CD3 expression in tumor region in all patients; **(B)** CD8-CD163 colocalization level in all patients; **(C)** Hotspot 1 CD3 expression in IIB and III stage patients; **(D)** CK-CD8 colocalization level in IIB and III stage patients.

In our study, we calculated the positive cell density for all biomarkers of immune cells (CD3, CD4, CD8, CD19, and CD163) in both the tumor region and IM, as described previously. We first analyzed the entire group of patients regardless of tumor stage; the results are presented in **Table 3**. In addition, some examples of survival curves are shown in **Figures 3A, B**. Specifically, Kaplan–Meier survival analysis revealed a significantly better survival of patients with high CD3+ (DFS, *P=*0.0014; OS, *P=*0.0031) and CD4+ (DFS, *P=*0.0101; OS, *P=*0.0106) signals in the tumor region, but not with biomarker in the IM. Taking the tumor region and IM together, high CD3+ (DFS, *P=*0.0014; OS, *P=*0.0031) and CD4+ (DFS, *P=*0.0101; OS, *P=*0.0106) signals were significantly correlated with better survival. For the Hotspot analysis, we found significantly better survival with higher CD3+ (DFS, *P=*0.0072; OS, *P=*0.0183) and CD4+ (DFS, *P=*0.0248; OS, *P=*0.0320) signals for Top1 density, higher CD3+ (DFS, *P=*0.0062; OS, *P=*0.0105) signals for Top2 density, and higher CD3+ (DFS, *P=*0.0062; OS, *P=*0.0105) and CD4+ (DFS, *P=*0.0439; OS, *P=*0.0522) signals for Top3 density.

**Table 3:** The univariate survival analysis of all parameters based on cell positive density for the group of all patients.

Parameters	DFS	OS	Parameters	DFS	OS	Parameters	DFS	OS
	p-value	p-value		p-value	p-value		p-value	p-value
TC-CD3	0.0014	0.0031	Hotspot1-CD3	0.0072	0.0183	CD3-CD4	0.0266	0.0206
TC-CD4	0.0101	0.0106	Hotspot1-CD4	0.0248	0.032	CD3-CD8	0.1522	0.1319
TC-CD8	0.8665	0.971	Hotspot1-CD8	0.2446	0.3365	CD3-CD19	0.835	0.992
TC-CD19	0.8845	0.9838	Hotspot1-CD19	0.8983	0.9839	CD3-CD163	0.5612	0.6578
TC-CD163	0.6284	0.4373	Hotspot1-CD163	0.8254	0.96	CD4-CD8	0.8326	0.9302
IM-CD3	0.1739	0.236	Hotspot2-CD3	0.0062	0.0105	CD4-CD19	0.4713	0.5612
IM-CD4	0.2137	0.228	Hotspot2-CD4	0.1161	0.1116	CD4-CD163	0.4028	0.3546
IM-CD8	0.2821	0.3729	Hotspot2-CD8	0.0961	0.1228	CD8-CD19	0.9734	0.8915
IM-CD19	0.5702	0.4635	Hotspot2-CD19	0.0834	0.0756	CD8-CD163	0.0193	0.0088
IM-CD163	0.785	0.9922	Hotspot2-CD163	0.8254	0.9496	CD19-CD163	0.4583	0.3515
TC+IM-CD3	0.0014	0.0031	Hotspot3-CD3	0.0062	0.0105	CK-CD3	0.0122	0.0177
TC+IM-CD4	0.0101	0.0106	Hotspot3-CD4	0.0439	0.0522	CK-CD4	0.2204	0.1843
TC+IM-CD8	0.4406	0.4996	Hotspot3-CD8	0.0693	0.0609	CK-CD19	0.4757	0.4015
TC+IM-CD19	0.8845	0.9838	Hotspot3-CD19	0.6974	0.6868	CK-CD163	0.7902	0.7039
TC+IM-CD163	0.6284	0.4373	Hotspot3-CD163	0.4092	0.5092	CK-CD8	0.3494	0.1933
						Immunoscore	0.2922	0.4089

DFS: Disease-free survival; OS: Overall survival; TC-CDx: The positive cell density of each biomarker in tumor core; IM-CDx: The positive cell density of each biomarker in invasive margin; TC+IM-CDx: The positive cell density of each biomarker in the region of tumor core and invasive margin together; Hotspotx-CDx: The positive cell density of first, second and third hotspot for each biomarker; CDx-CDx: The MH index between each pair of biomarkers; CK-CDx: The MH index between tumor cells and each biomarker.

For patients with stage IIB and III cancer, a significant better survival was observed with higher CD4+ (DFS, *P=*0.0155; OS, *P=*0.0121) signals in the tumor region, and CD3+ (DFS, *P=*0.0110; OS, *P=*0.0100) and CD8+ (DFS, *P=*0.0420; OS, *P=*0.0809) signals in the IM, which is shown in **Table 4**. For the tumor region and IM together, higher CD4+ (DFS, *P=*0.0156; OS, *P=*0.0121) and CD19+ (DFS, *P=*0.0206; OS, *P=*0.0309) signals were significantly correlated with better survival. In the Hotspot analysis, we found a significantly better survival with CD3+ (DFS, *P=*0.0026; OS, *P=*0.0060) signals for Top1 density; CD3+ (DFS, *P=*0.0005; OS, *P=*0.0004), CD8+ (DFS, *P=*0.0331; OS, *P=*0.0728), and CD19+ (DFS, *P=*0.0027; OS, *P=*0.0053) signals for Top2 density; and CD3+ (DFS, *P=*0.0005; OS, *P=*0.0004), CD4+ (DFS, *P=*0.0213; OS, *P=*0.0728), CD8+ (DFS, *P=*0.0331; OS, *P=*0.0728), and CD19+ (DFS, *P=*0.0008; OS, *P=*0.0020) signals for Top3 density.

**Table 4:** The survival analysis of all parameters based on cell positive density for the group of IIB and III stage patients.

Parameters	DFS p-value	OS p-value	Parameters	DFS p-value	OS p-value	Parameters	DFS p-value	OS p-value
TC-CD3	0.3303	0.4310	Hotspot1-CD3	0.0026	0.0060	CD3-CD4	0.6576	0.8168
TC-CD4	0.0156	0.0121	Hotspot1-CD4	0.0692	0.1067	CD3-CD8	0.0939	0.0854
TC-CD8	0.1147	0.1781	Hotspot1-CD8	0.6372	0.3687	CD3-CD19	0.3660	0.3705
TC-CD19	0.1426	0.1625	Hotspot1-CD19	0.0843	0.0968	CD3-CD163	0.1017	0.1103
TC-CD163	0.8636	0.6004	Hotspot1-CD163	0.3582	0.6605	CD4-CD8	0.8451	0.7202
IM-CD3	0.0110	0.0100	Hotspot2-CD3	0.0005	0.0004	CD4-CD19	0.8994	0.8959
IM-CD4	0.3308	0.3487	Hotspot2-CD4	0.0692	0.1067	CD4-CD163	0.0635	0.0770
IM-CD8	0.0420	0.0809	Hotspot2-CD8	0.0331	0.0728	CD8-CD19	0.3173	0.3040
IM-CD19	0.1747	0.2119	Hotspot2-CD19	0.0027	0.0053	CD8-CD163	0.0161	0.0190
IM-CD163	0.9659	0.7473	Hotspot2-CD163	0.3582	0.6605	CD19-CD163	0.1180	0.1293
TC+IM-CD3	0.3303	0.4310	Hotspot3-CD3	0.0005	0.0004	CK-CD3	0.0580	0.0465
TC+IM-CD4	0.0156	0.0121	Hotspot3-CD4	0.0213	0.0728	CK-CD4	0.0031	0.0037
TC+IM-CD8	0.2256	0.2946	Hotspot3-CD8	0.0331	0.0728	CK-CD19	0.1861	0.1330
TC+IM-CD19	0.0206	0.0309	Hotspot3-CD19	0.0008	0.0020	CK-CD163	0.5274	0.5851
TC+IM-CD163	0.8636	0.6004	Hotspot3-CD163	0.3582	0.6605	CK-CD8	0.0444	0.0152
						Immunoscore	0.0634	0.0849

DFS: Disease-free survival; OS: Overall survival; TC-CDx: The positive cell density of each biomarker in tumor core; IM-CDx: The positive cell density of each biomarker in invasive margin; TC+IM-CDx: The positive cell density of each biomarker in the region of tumor core and invasive margin together; Hotspotx-CDx: The positive cell density of first, second and third hotspot for each biomarker; CDx-CDx: The MH index between each pair of biomarkers; CK-CDx: The MH index between tumor cells and each

### 3.3 Correlation between spatial parameters and prognostic survival

The MH index was computed, and survival analysis was performed to determine the correlation between spatial parameters and prognostic survival. For all patients, higher MH indices of CD3-CD4 (DFS, *P=*0.0266; OS, *P=*0.0206), CD8-CD163 (DFS, *P=*0.0193; OS, *P=*0.0088), and CK-CD3 (DFS, *P=*0.0122; OS, *P=*0.0177) were significantly correlated with better survival (**Table 3**; **Figure 3C**).

For patients with more advanced cancer (stages IIB and III), the MH index between CD8+ and CD163+ cells were significantly correlated with survival (DFS, *P=*0.0161; OS, *P=*0.0190). Higher colocalization of tumor cells (CK) and some biomarkers was significantly correlated with better survival, such as CD3+ (DFS, *P=*0.0580; OS, *P=*0.0465), CD4+ (DFS, *P=*0.0031; OS, *P=*0.0037), and CD8+ (DFS, *P=*0.0444; OS, *P=*0.0152, **Table 4**; **Figure 3D**).

### 3.4 Correlation between Immunoscore and prognostic survival

The Immunoscore was calculated using CD3+ and CD8+ densities in the tumor region and IM, as described previously. For all patients in our study, we did not find a significant correlation between the Immunoscore and survival (DFS, *P=*0.2922; OS, *P=*0.4089, **Table 3**). The relationship between the Immunoscore for patients with stage IIB and III disease and survival was also not significant, but the p-values were very close to 0.05 (DFS, *P=*0.0634; OS, *P=*0.0849, **Table 4**).

### 3.5 Multivariate analysis

Since there were only 29 patients in the late-stage group (stage IIB and III), multivariate analysis was performed for all patient groups, as shown in **Tables 5**, **6**. Tumor size, age, tumor stage, basal-like breast cancer, and Ki67 and p53 expression were considered independent variables that were significantly correlated with patient survival. For DFS, Ki67 expression was the only parameter that was significant in the multivariate survival analysis. For OS, the colocalization of CD8 and CD163 remained significantly correlated with better survival (*P*=0.02, **Table 6**).

**Table 5:** The multivariate survival analysis with DFS of all parameters based on cell positive density for the group of all patients.

p-values for parameters	P value
Age	0.54
Basal-like	0.83
Stage	0.75
Tumor size	0.02
grade	0.13
ki67% (Threshold = 14)	0.01
P53	0.91
TC-CD3	0.65
TC-CD4	0.89
TC+IM-CD3	0.65
TC+IM-CD4	0.89
Hotspot1-CD3	0.65
Hotspot1-CD4	0.16
Hotspot2-CD3	0.92
Hotspot3-CD3	0.92
Hotspot3-CD4	0.28
CD3-CD4	0.45
CD8-CD163	0.35
CK-CD3	0.84

**Table 6:** The multivariate survival analysis with OS of all parameters based on cell positive density for the group of all patients.

p-values for parameters	P value
Age	0.85
Basal-like	0.46
Stage	0.39
Tumor size	0.03
grade	0.21
ki67% (Threshold = 14)	0.05
P53	0.92
TC-CD3	0.8
TC-CD4	0.97
TC+IM-CD3	0.8
TC+IM-CD4	0.97
Hotspot1-CD3	0.29
Hotspot1-CD4	0.67
Hotspot2-CD3	0.98
Hotspot3-CD3	0.98
CD3-CD4	0.16
CD8-CD163	0.02
CK-CD3	0.74

## 4 Discussion

The immune context of TNBC has gained acceptance as an important clinical correlate, raising the possibility that modulating immune responses *via* immunotherapy will constitute an effective therapeutic strategy. However, only 8–20% of preselected patients with TNBC benefit from anti-programmed cell death-1 ligand 1 (PD-L1) or anti-programmed death protein-1 (PD-1) immunotherapy, highlighting the need for a better understanding of how the TIME architecture influences outcomes in TNBC and responses to current treatment modalities (16, 17). It is necessary to establish cohort data and reveal the specific mechanisms and interactions of the TIME components. IHC is commonly used as a diagnostic technique in the field of histopathology, but usually only one biomarker can be detected in each pathology section in routine pathological examinations, which is a limitation in parsing the TIME of clinical cases. Several extended testing platforms based on RNA sequencing, immunofluorescence, and mass spectrometry have emerged to provide a comprehensive landscape of the TIME composition and marker distribution to uncover the pathogenesis of complex disorders (18–20). However, these platforms can be costly.

In this study, we used computational imaging analysis of multiple biomarkers based on IHC images commonly used in routine pathology testing. We investigated the expression of five different immune markers in different areas of the tumor region, thus providing a deeper understanding of complex TIMEs by combining immune cell identification and localization in matched clinical samples. The specially prepared serial sections were well-aligned and combined for analysis. Because the same primary antibody and 3,3′-diaminobenzidine (DAB) systems were used throughout the experiment, the staining was highly consistent. In addition to the reagents and equipment for clinical examination, only an additional computer server was needed for rapid analysis.

Image registration is a widely used technique in medical imaging (21), but it is not popular in studies of pathological images. Scanning image analysis with the help of image registration and the spatial positions of IHC images with multiple markers are correlated. Registration algorithms increasingly support digital pathology research. In a previous study, serial sections of breast tissue samples were aligned, enabling the user to scroll through the sections in a single viewer. The entire computational workflow starts with image processing. We use cell nucleus segmentation to obtain the location information of labeled cells in their original slides and perform image registration using a multimodal protocol to calculate transformation matrices that map all slides to the reference CK slide. We aligned the images based on combined serial sections and determined the spatial information of multiple biomarkers (22). After image scanning and registration, the expression status of multiple markers, including density and location, can be integrated into a single virtual image. As a result, subsequent nano-analysis, such as confocal analysis and hot spot analysis, can be performed more easily and accurately on the computer. IHC staring and slide scanners are commonly used in general pathology, and image registration is a well-established technique in medical imaging. Therefore, this multidisciplinary method is a practical aid for the analysis of a large number of markers.

The spatial information of biomarkers in the tumor microenvironment has garnered more attention from researchers. In 2014, Angelo et al. found that the spatial distribution of immune and tumor cells influences immune activity and is associated with patient survival (23). In our study, we focused on the spatial information of immune cells within the tumor regions and attempted to determine the colocalization of specific immune cell types or the colocalization of tumor cells and different immune cell types using the MH index.

Among all regions, CD163+ macrophages were evenly distributed in both the IM and TC. Almost twice as many CD3, CD4, CD8, and CD19 lymphocytes were found in the IM group than in the TC group. This is consistent with previous findings (24, 25). Of the five markers, we found that CD3 and CD4 had the greatest influence on patient prognosis, and this influence had little relationship with spatial distribution of the markers in tumors. This is consistent with previous results showing that high CD3+ cell density is associated with a favorable outcome in oropharyngeal cancer, and a low CD3+ cell count has been shown to predict shorter DFS in patients with colon and cervical cancer (26, 27). There have also been reports that CD3 and CD4 are biomarkers that predict pathologic complete response (pCR) to neoadjuvant chemotherapy in TNBC (28, 29). Few studies have investigated the prognostic significance of CD3 and CD4 alone and their distribution in TNBC. However, a previous study showed high heterogeneity in CD3 and CD4 distributions (25). Our spatial analysis showed that there were significant differences between the ER and LR groups and between the DM and non-DM groups in the density of CD3+ or CD4+ immune cells in all tumor regions, including the TC, IM, combined tumor region, and all three hotspots. This is supported by previous studies that have reported that T cells in all tumoral regions are associated with improved prognosis (30). In more advanced (stage IIB and III) TNBC cases, CD3+ cells in the IM and hotspots were more suggestive of patient prognosis than CD3+ cells in the TC and combined tumor regions. This showed that T-cell accumulation and especially location (in IM) had more tumor effect than scattered distribution in TC. It is consistent that the tumor cells in IM were more aggressive than those in TC (31). To the best of our knowledge, we are the first to report the prognostic effects of CD3 and CD4 in TNBC and their spatial relevance.

In general, a high number of CD8+ TILs are associated with increased DFS and OS in breast cancer (32, 33). Both CD8 mRNA and protein expression have been evaluated, and both were predictive of survival in TNBC (34, 35). In our cohort, the CD8+ cells did not show a significant difference between the ER and LR groups, as well as between the DM and non-DM groups, and neither showed any significant effect on patient prognosis in all stages of TNBC. However, we found that in late-stage TNBC, CD8+ cells were significantly associated with patient prognosis. This effect was related to the localization of the CD8+ cells. Patients with high CD8+ cell signals in the IM and hotspots had significantly longer DFS and OS times. Some research with large sample size had proven that CD8 positive T cell was significantly associated with better prognosis (34). Our result was consistent with the previous research, except that no significant p-value was obtained, which may be due to the limited sample size. Another reason may be that there were few reports on the relationship between CD8 expression and tumor TNM stages. From another point of view, our results reflect that the effect of CD8-positive cells was more obvious in more advanced stages of TNBC. To the best of our knowledge, we are also the first to report a relationship between the spatial localization of CD8+ cells and patient survival.

The effect of B cells on patient prognosis in TNBC has been brought to the spotlight of cutting-edge of researching recently. The CD20 score is known to predict pCR independently of age, size, nuclear grade, nodal status, and expression of estrogen receptor (ER), progesterone receptor (PR), human epidermal growth factor receptor 2 (HER2), and Ki67 (28). Some researchers found that tumor-infiltrating B lymphocytes were significantly associated with better prognosis in TNBC (36, 37). However, not many other studies have showed any relationship between B cells and TNBC prognosis (25). CD19 is a marker of active B cells. Therefore, we detected CD19 instead of CD20. Through analysis of the spatial expression of CD19, we found that B cell densities were significantly different between the ER and LR groups, but not between the DM and non-DM groups, but there was no association with DFS and OS. The differences were more obvious in the TC, TC+IM, and hotspots than in IM alone.

Colocalization analysis found that some interesting combinations, such as a high density of CD3+/CD4+ and CD3+/CK+ cells, were associated with significantly longer DFS and OS times at all TNBC stages. In late-stage cases, all T cell markers (CD3, CD4, and CD8) and CK colocalization indicated a better prognosis. To our knowledge, this is the first study to demonstrate the importance of CK and T cell colocalization. We also found that CD8+/CD163+ colocalization significantly extended the DFS and OS of TNBC cases overall and specifically in more advanced cases (stage IIB and III). There were significant differences in CD8+/CD163+ cells between the ER and LR groups, and between the DM and non-DM groups. Multivariate survival analysis also showed that CD8+/CD163+ colocalization was an independent prognostic factor. There have been several studies on CD8 and CD163 expression in TNBC. Jamiyan et al. reported that the infiltration of CD163+ tumor-associated macrophages in both the tumor stroma and tumor nest was associated with poor patient prognosis in TNBC (38). Foulds et al. showed that high expression of CD163 in the peripheral blood of patients with TNBC predicted relapse-free survival (39). Infiltration of CD8+ T lymphocytes into solid tumors is associated with a good prognosis in various types of cancer, including TNBC (32, 35, 40). Fortis et al. further found that the densities of CD8+ and CD163+ cells were different in the TC and IM, and the combined evaluation of both compartments was significantly related to breast cancer survival (41). Our experiments also indicated the importance of the spatial distribution of CD8 and CD68 and showed that colocalization of CD8 and CD68 independently predicted good outcomes.

We also calculated the Immunoscore of each patient. In colorectal carcinoma, the Immunoscore was shown to be a marker that surpassed the traditional gold standard of diagnostics using tumor stage, lymph node swelling, and metastatic invasion (42). However, we did not find a significant correlation between this parameter and patient survival. However, it is worth noting that the p-values of the correlation between the survival and Immunoscore of patients in stages IIB and III were very close to 0.05 (DFS, *P=*0.0634; OS, *P=*0.0849). We noticed that there were only 29 patients with stage IIB and III disease. If we had collected samples from more patients in these stages, we could have probably observed the prognostic value of Immunoscore in this group of patients. Immunoscore applications in breast cancer require more research with a larger cohort.

Compared with other techniques related to spatial analysis (multiplex immunofluorescence images or multiplex IHC staining), computational imaging analysis has the advantages of lower costs and higher data accuracy (the entire tissue section is scanned), and thus has a greater possibility of clinical applications. We realize that pathological slides cannot be aligned precisely for each cell, owing to the limitations of existing technology. However, if we focus on the expression of biomarkers in tissue regions, we can still analyze the tumor microenvironment. In future studies, more biomarkers, not only those identifying immune cells will be analyzed.

In summary, we applied the image registration technique to pathological image research and attempted to assess multiplex immunofluorescence images of serial sections and IHC staining at the level of region alignment. Some biomarkers (such as CD3 and CD4) and the colocalization of immune cells (such as the colocalization of CD8 and CD163, or tumor cells and CD3/CD4/CD8) showed a significant association with patient survival, which revealed the prognostic value of the tumor microenvironment and the potential application of image registration to breast cancer prognostics. In view of the limited sample size of this study, follow-up investigation are planned to better confirm the potential prognostic role of tumor infiltrating lymphocytes by enlarging the sampled population with further clinical validation.

## Data availability statement

The raw data supporting the conclusions of this article will be made available by the authors, without undue reservation.

## Ethics statement

This study was approved by Peking Union Medical College Hospital Institutional Review Board (PUMCH IRB). All procedures performed in this study involving human participants were in accordance with the ethical standards of the institutional and national research committee and with the Declaration of Helsinki and its later amendments. The patients/participants provided their written informed consent to participate in this study.

## Author contributions

XR: conceptualization, methodology, formal analysis, writing, editing, and reviewing. YS: conceptualization, methodology, investigation, data curation, and writing. JP and LC: methodology, investigation, and resources. ZL: conceptualization, methodology, reviewing, funding acquisition, and supervision. HW: conceptualization, methodology, reviewing and project administration. All authors contributed to the article and approved the submitted version.
