# Supplementary material for: Prognostic value of various immune cells and Immunoscore in triple-negative breast cancer
Source: Front Immunol. 2023 Apr 6;14:1137561. doi: 10.3389/fimmu.2023.1137561 (PMC10117828; doi:10.3389/fimmu.2023.1137561)
Supplement: Supplementary file 1 [file Table_1.docx]

Supplementary Table1

| Antibody | Clone | Dilution | Source | Positive style | Positive control | Heat-induced Antigen Retrieval | Incubation |
| --- | --- | --- | --- | --- | --- | --- | --- |
| CD3 | Mouse Monoclonal  LN10 | Prediluted | Leica | Membrane and Cytoplasm | tonsil | 100°C  20 min | 25°C  15 min |
| CD4 | Mouse Monoclonal  4B12 | Prediluted | Leica | Membrane | tonsil | 100°C  15 min | 25°C  15 min |
| CD8 | Mouse Monoclonal  4B11 | Prediluted | Leica | Membrane | tonsil | 100°C  20 min | 25°C  15 min |
| CD19 | rabbit monoclonal  BP6046 | Prediluted | Bailing | Membrane and Cytoplasm | tonsil | 100°C  20 min | 25°C  15 min |
| CD163 | Mouse Monoclonal  10D6 | Prediluted | ZSGB-BIO | Cytoplasm | tonsil | 100°C  20 min | 25°C  30 min |
| AE1/AE3 | Mouse Monoclonal  AE1/AE3 | Prediluted | DAKO | Cytoplasm | tonsil | 98°C  30 min | 25°C  30 min |
